# {6,6′-Dimeth­oxy-2,2′-[naphthalene-2,3-diylbis(nitrilo­methyl­idyne)]diphenolato}thio­cyanato­cobalt(III) diethyl ether dichloro­methane solvate

**DOI:** 10.1107/S1600536809000841

**Published:** 2009-02-06

**Authors:** Zhong Yu, Takayoshi Kuroda-Sowa, Atsuhiro Nabei, Masahiko Maekawa, Takashi Okubo

**Affiliations:** a Department of Chemistry, Faculty of Science and Engineering, Kinki University, Higashi-Osaka, Osaka 577-8502, Japan; b Department of Applied Chemistry, School of Science, Xi’an University of Technology, Xi’an 710048, People’s Republic of China

## Abstract

In the title complex, [Co(C_26_H_20_N_2_O_4_)(NCS)]·C_4_H_10_O·CH_2_Cl_2_, the penta­coordinated Co^III^ atom exhibits a distorted square-pyramidal geometry with an *N*,*N*′,*O*,*O*′ tetra­dentate Schiff base ligand in the basal plane and one thio­cyanate ligand at the apical site. The diethyl ether mol­ecule is located in a cavity provided by four O atoms of the ligand with weak C—H⋯O inter­actions, generating two short O⋯O contact distances [2.766 (3) and 2.745 (3) Å] between the diethyl ether mol­ecule and the ligand. The crystal structure is stabilized by the weak C—H⋯O and C—H⋯N inter­actions and π–π inter­actions between the naphthyl ring system and the benzene ring [centroid–centroid distance = 3.657 (5) Å] and between the two naphthyl ring systems [centroid–centroid distance = 4.305 (2) Å].

## Related literature

For the properties of Co(III) complexes with Schiff base ligands, see: Ito & Katsuki (1999 ▶); Wezenberg & Kleij (2008 ▶); Di Bella *et al.* (1995 ▶). For related structures, see: Kennedy *et al.* (1984 ▶); Marzilli *et al.* (1985 ▶); Álvarez *et al.* (2002 ▶). For hydrogen-bond length data, see: Desiraju & Steiner (1999 ▶). For non-bonded contact distances, see: Rowland & Taylor (1996 ▶); De Angelis *et al.* (1996 ▶). For the preparation of bis­(*o*-vanillin)-2,3-naphthalene­diimine, see: Nabei *et al.* (2008 ▶).
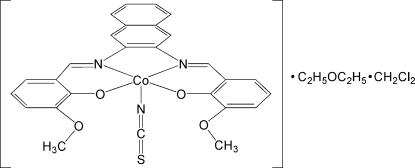


## Experimental

### 

#### Crystal data

[Co(C_26_H_20_N_2_O_4_)(NCS)]·C_4_H_10_O·CH_2_Cl_2_
*M* *_r_* = 700.52
Monoclinic, 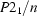

*a* = 9.1935 (9) Å
*b* = 13.3640 (11) Å
*c* = 25.910 (3) Å
β = 92.462 (6)°
*V* = 3180.4 (5) Å^3^
*Z* = 4
Mo *K*α radiation
μ = 0.82 mm^−1^
*T* = 120 (1) K
0.40 × 0.10 × 0.10 mm

#### Data collection

Rigaku Mercury diffractometer
Absorption correction: multi-scan (Jacobson, 1998 ▶) *T*_min_ = 0.799, *T*_max_ = 0.921
24340 measured reflections
7241 independent reflections
6234 reflections with *I* ^2^ > 2σ(*I* ^2^)
*R*_int_ = 0.051

#### Refinement

*R*[*F* ^2^ > 2σ(*F* ^2^)] = 0.075
*wR*(*F* ^2^) = 0.136
*S* = 1.21
7241 reflections
397 parameters
H-atom parameters constrained
Δρ_max_ = 0.69 e Å^−3^
Δρ_min_ = −0.60 e Å^−3^

### 

Data collection: *CrystalClear* (Rigaku, 2001 ▶); cell refinement: *CrystalClear*; data reduction: *CrystalStructure* (Rigaku/MSC, 2007 ▶); program(s) used to solve structure: *SIR97* (Altomare *et al.*, 1999 ▶); program(s) used to refine structure: *SHELXL97* (Sheldrick, 2008 ▶); molecular graphics: *SHELXL97*; software used to prepare material for publication: *CrystalStructure*.

## Supplementary Material

Crystal structure: contains datablocks global, I. DOI: 10.1107/S1600536809000841/is2372sup1.cif

Structure factors: contains datablocks I. DOI: 10.1107/S1600536809000841/is2372Isup2.hkl

Additional supplementary materials:  crystallographic information; 3D view; checkCIF report

## Figures and Tables

**Table 1:** Hydrogen-bond geometry (Å, °)

*D*—H⋯*A*	*D*—H	H⋯*A*	*D*⋯*A*	*D*—H⋯*A*
C1—H1⋯N3^i^	0.95	2.64	3.579 (4)	172
C28—H28*A*⋯O2	0.99	2.42	3.352 (4)	157
C29—H29*B*⋯O4	0.99	2.94	3.424 (4)	111
C30—H30*B*⋯O3	0.98	2.96	3.607 (5)	125
C32—H32*B*⋯O2	0.98	2.80	3.453 (4)	124
C32—H32*C*⋯O1	0.98	2.80	3.423 (4)	122

Symmetry code: (i) 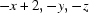.

**Fig. 1.**
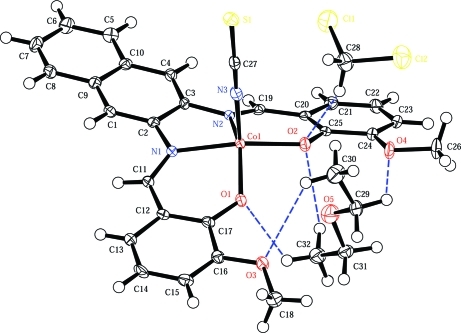
A view of the title molecule, showing the atom-labelling scheme. Displacement ellipsoids are drawn at the 50% probability level. Weak hydrogen bonds are indicated with dashed lines.

**Fig. 2.**
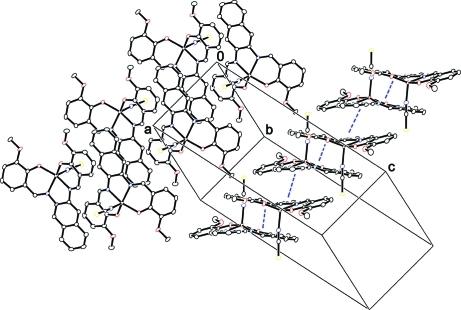
A view of crystal packing of the title complex. The π-π interactions are indicated with dashed lines. For clarity, H atoms are not shown.

## supplementary crystallographic information

### Comment

Cobalt Schiff base complexes have undergone extensive research as a promising catalyst for various homogeneous reactions (Ito & Katsuki, 1999). Since novel solid state properties on structural types, conductive and magnetic properties (Wezenberg & Kleij, 2008; Di Bella *et al.*, 1995), they recently attract new attentions on the material applications. Herein we report a new Co(III) complex based on the Schiff base ligand bis(*o*-vanillin)-2,3-naphthalenediimine.

In the title complex, the Co(III) ion shows the five-coordinated square pyramidal geometry, which is defined by two N and two O atoms of the tetradentate ligand in the approximate basal plane and one N atoms of thiocyanate in the apical position (Fig. 1). The bond distances and angles associated with Co(III) atoms are comparable with related five-coordinated cobalt species (Kennedy *et al.*, 1984; Marzilli *et al.*, 1985; Álvarez *et al.*, 2002). The ligand plane is distorted with a dihedral angle of 27.81 (12)° between two phenyl rings. The diethyl ether molecule is approximately perpendicular to ligand plane, with the O atom almost coplanar in the ligand.

In the crystal structure, the complex molecule provides a planar cavity of four O atoms which accommodates a diethyl ether molecule *via* weak C—H···O interactions (Table 1). The range for the H···O distances agree with those found for weak C—H···O hydrogen bonds (Desiraju & Steiner, 1999). There are short non-bonded intramolecular distances between O atoms of diethyl ether and ligand: O1···O5 = 2.766 (3) Å and O2···O5 = 2.745 (3) Å, slightly less than the corresponding van der Waals distances (O···O = 2.80 Å; Rowland & Taylor, 1996). It may be attributed to those weak interactions between diethyl ether and complex, as well as some effects of crystal packing, which is comparable with a distance [Na···O(Me) = 2.54 (3) Å] in a similar structure (De Angelis *et al.*, 1996). The crystal structure is further stabilized by additional interactions C1—H1···N3^i ^and C28—H28A···O2 (Table 2), together with extended π-π interactions between naphthyl rings and phenyl rings [centroid-centroid^i ^distances of 3.657 (5) Å, dihedral angles of 12.97 (10)°] as well as naphthyl rings [centroid-centroid^ii^ distances of 4.305 (2) Å, interplanar distances of 3.521 (4) Å] of adjacent molecules [symmetry codes: (i) -*x* + 2, -*y*, -*z*; (ii) -*x* + 1, -*y*, -*z*], forming an infinite three-dimensional network (Fig. 2).

### Experimental

The desired ligand, bis(*o*-vanillin)-2,3-naphthalenediimine, was synthesized according to the literature procedures (Nabei *et al.*, 2008). A solution of Co(SCN)_2_ (0.1 mmol, 17.6 mg) in methanol (10 ml) was layered over a solution of ligand (0.1 mmol, 42.6 mg) in dichloromethane (10 ml). After standing for two weeks at room temperature, the brown brick crystals of title complex suitable for X-ray analysis were obtained.

### Refinement

All H atoms were placed in calculated positions and refined as riding, with C—H = 0.95–0.99 Å and *U*_iso_(H) = 1.2*U*_eq_(C).

### Crystal data

**Table:**

[Co(C_26_H_20_N_2_O_4_)(NCS)]·C_4_H_10_O·CH_2_Cl_2_	*F*(000) = 1448.00
*M**_r_* = 700.52	*D*_x_ = 1.463 Mg m^−^^3^
Monoclinic, *P*2_1_/*n*	Mo *K*α radiation, λ = 0.71070 Å
Hall symbol: -P 2yn	Cell parameters from 8164 reflections
*a* = 9.1935 (9) Å	θ = 3.0–27.5°
*b* = 13.3640 (11) Å	µ = 0.82 mm^−^^1^
*c* = 25.910 (3) Å	*T* = 120 K
β = 92.462 (6)°	Block, brown
*V* = 3180.4 (5) Å^3^	0.40 × 0.10 × 0.10 mm
*Z* = 4	

### Data collection

**Table:**

Rigaku Mercury diffractometer	6234 reflections with *F*^2^ > 2σ(*F*^2^)
Detector resolution: 7.31 pixels mm^-1^	*R*_int_ = 0.051
ω scans	θ_max_ = 27.5°
Absorption correction: multi-scan (Jacobson, 1998)	*h* = −11→11
*T*_min_ = 0.799, *T*_max_ = 0.921	*k* = −17→16
24340 measured reflections	*l* = −33→33
7241 independent reflections	

### Refinement

**Table:**

Refinement on *F*^2^	0 restraints
*R*[*F*^2^ > 2σ(*F*^2^)] = 0.075	H-atom parameters constrained
*wR*(*F*^2^) = 0.136	*w* = 1/[σ^2^(*F*_o_^2^) + (0.0406*P*)^2^ + 5.4074*P*] where *P* = (*F*_o_^2^ + 2*F*_c_^2^)/3
*S* = 1.21	(Δ/σ)_max_ < 0.001
7241 reflections	Δρ_max_ = 0.69 e Å^−^^3^
397 parameters	Δρ_min_ = −0.60 e Å^−^^3^

### Special details

**Table:**

Geometry. ENTER SPECIAL DETAILS OF THE MOLECULAR GEOMETRY
Refinement. Refinement was performed using all reflections. The weighted *R*-factor (*wR*) and goodness of fit (*S*) are based on *F*^2^. *R*-factor (gt) are based on *F*. The threshold expression of *F*^2^ > 2.0 σ(*F*^2^) is used only for calculating *R*-factor (gt).

### Fractional atomic coordinates and isotropic or equivalent isotropic displacement parameters (Å^2^)

**Table:**

	*x*	*y*	*z*	*U*_iso_*/*U*_eq_	
Co1	0.86917 (5)	0.14242 (3)	0.104634 (16)	0.01291 (12)	
Cl1	0.94249 (14)	−0.01789 (9)	0.26072 (5)	0.0457 (3)	
Cl2	1.08816 (13)	0.13888 (10)	0.32166 (4)	0.0432 (2)	
S1	1.23023 (10)	−0.10089 (7)	0.16110 (3)	0.0209 (2)	
O1	0.9600 (2)	0.26531 (17)	0.07695 (9)	0.0169 (5)	
O2	0.8278 (2)	0.20652 (17)	0.17258 (9)	0.0169 (5)	
O3	1.1184 (2)	0.42651 (18)	0.06792 (9)	0.0208 (5)	
O4	0.8313 (2)	0.30348 (19)	0.25937 (9)	0.0227 (5)	
O5	0.9766 (3)	0.3836 (2)	0.16464 (11)	0.0342 (7)	
N1	0.8470 (3)	0.0896 (2)	0.02833 (10)	0.0136 (5)	
N2	0.6785 (3)	0.0629 (2)	0.10650 (10)	0.0125 (5)	
N3	1.0299 (3)	0.0455 (2)	0.12721 (11)	0.0200 (6)	
C1	0.7473 (3)	−0.0599 (2)	−0.01750 (12)	0.0143 (6)	
C2	0.7520 (3)	0.0062 (2)	0.02329 (13)	0.0135 (6)	
C3	0.6577 (3)	−0.0073 (2)	0.06516 (12)	0.0136 (6)	
C4	0.5599 (3)	−0.0851 (2)	0.06453 (13)	0.0152 (6)	
C5	0.4501 (3)	−0.2336 (2)	0.02066 (14)	0.0212 (7)	
C6	0.4441 (4)	−0.2974 (2)	−0.02095 (14)	0.0231 (8)	
C7	0.5377 (4)	−0.2840 (2)	−0.06230 (15)	0.0237 (8)	
C8	0.6372 (3)	−0.2082 (2)	−0.06120 (13)	0.0188 (7)	
C9	0.6461 (3)	−0.1400 (2)	−0.01923 (13)	0.0159 (6)	
C10	0.5509 (3)	−0.1530 (2)	0.02226 (13)	0.0155 (6)	
C11	0.8959 (3)	0.1312 (2)	−0.01225 (12)	0.0151 (6)	
C12	0.9881 (3)	0.2190 (2)	−0.01257 (13)	0.0151 (6)	
C13	1.0523 (3)	0.2418 (2)	−0.05992 (13)	0.0185 (7)	
C14	1.1415 (4)	0.3229 (2)	−0.06411 (14)	0.0224 (8)	
C15	1.1658 (4)	0.3869 (2)	−0.02166 (14)	0.0202 (7)	
C16	1.1014 (3)	0.3677 (2)	0.02430 (13)	0.0175 (7)	
C17	1.0133 (3)	0.2812 (2)	0.03134 (13)	0.0156 (7)	
C18	1.2007 (4)	0.5168 (2)	0.06299 (15)	0.0267 (8)	
C19	0.5787 (3)	0.0745 (2)	0.14003 (13)	0.0140 (6)	
C20	0.5903 (3)	0.1340 (2)	0.18630 (12)	0.0144 (6)	
C21	0.4719 (3)	0.1279 (2)	0.21954 (13)	0.0176 (7)	
C22	0.4720 (4)	0.1784 (2)	0.26559 (13)	0.0207 (7)	
C23	0.5933 (4)	0.2381 (2)	0.28035 (13)	0.0193 (7)	
C24	0.7092 (3)	0.2458 (2)	0.24873 (13)	0.0165 (7)	
C25	0.7134 (3)	0.1936 (2)	0.20060 (12)	0.0141 (6)	
C26	0.8445 (4)	0.3472 (3)	0.30996 (14)	0.0286 (9)	
C27	1.1133 (3)	−0.0151 (2)	0.14180 (13)	0.0163 (7)	
C28	1.0497 (4)	0.0897 (3)	0.25913 (15)	0.0294 (9)	
C29	1.1140 (4)	0.4060 (2)	0.19570 (14)	0.0223 (7)	
C30	1.2196 (4)	0.3211 (3)	0.19249 (17)	0.0334 (9)	
C31	0.8751 (4)	0.4713 (2)	0.16089 (15)	0.0229 (8)	
C32	0.7414 (4)	0.4437 (3)	0.12821 (15)	0.0258 (8)	
H1	0.8123	−0.0517	−0.0447	0.017*	
H4	0.4977	−0.0935	0.0926	0.018*	
H5	0.3870	−0.2434	0.0483	0.025*	
H6	0.3763	−0.3511	−0.0220	0.028*	
H7	0.5312	−0.3281	−0.0911	0.028*	
H8	0.7011	−0.2010	−0.0888	0.023*	
H11	0.8691	0.1019	−0.0447	0.018*	
H13	1.0330	0.2002	−0.0891	0.022*	
H14	1.1870	0.3358	−0.0957	0.027*	
H15	1.2269	0.4438	−0.0247	0.024*	
H18A	1.2052	0.5524	0.0961	0.032*	
H18B	1.1536	0.5595	0.0364	0.032*	
H18C	1.2996	0.5003	0.0531	0.032*	
H19	0.4893	0.0403	0.1333	0.017*	
H21	0.3902	0.0877	0.2096	0.021*	
H22	0.3916	0.1732	0.2873	0.025*	
H23	0.5950	0.2731	0.3123	0.023*	
H26A	0.9345	0.3864	0.3132	0.034*	
H26B	0.8471	0.2941	0.3361	0.034*	
H26C	0.7609	0.3910	0.3152	0.034*	
H28A	0.9985	0.1411	0.2377	0.035*	
H28B	1.1423	0.0737	0.2428	0.035*	
H29A	1.1586	0.4679	0.1825	0.027*	
H29B	1.0910	0.4173	0.2322	0.027*	
H30A	1.3087	0.3372	0.2130	0.040*	
H30B	1.2433	0.3106	0.1564	0.040*	
H30C	1.1760	0.2602	0.2061	0.040*	
H31A	0.9247	0.5289	0.1452	0.027*	
H31B	0.8466	0.4911	0.1959	0.027*	
H32A	0.6753	0.5012	0.1257	0.031*	
H32B	0.6920	0.3874	0.1441	0.031*	
H32C	0.7700	0.4247	0.0936	0.031*	

### Atomic displacement parameters (Å^2^)

**Table:**

	*U*^11^	*U*^22^	*U*^33^	*U*^12^	*U*^13^	*U*^23^
Co1	0.0163 (2)	0.0129 (2)	0.0096 (2)	−0.00135 (19)	0.00109 (17)	0.00003 (18)
Cl1	0.0561 (7)	0.0395 (6)	0.0431 (6)	−0.0178 (5)	0.0187 (5)	−0.0048 (5)
Cl2	0.0424 (6)	0.0508 (7)	0.0355 (6)	0.0001 (5)	−0.0067 (5)	−0.0069 (5)
S1	0.0193 (4)	0.0229 (4)	0.0203 (4)	0.0029 (3)	−0.0010 (3)	0.0034 (3)
O1	0.0238 (13)	0.0165 (12)	0.0107 (11)	−0.0038 (10)	0.0018 (9)	−0.0011 (9)
O2	0.0203 (12)	0.0179 (12)	0.0128 (11)	−0.0037 (10)	0.0036 (9)	−0.0025 (9)
O3	0.0301 (14)	0.0181 (12)	0.0142 (12)	−0.0102 (11)	−0.0006 (10)	−0.0005 (9)
O4	0.0307 (14)	0.0230 (13)	0.0145 (12)	−0.0071 (11)	0.0018 (10)	−0.0085 (10)
O5	0.0363 (17)	0.0322 (16)	0.0337 (16)	−0.0014 (13)	−0.0025 (13)	−0.0020 (12)
N1	0.0176 (14)	0.0122 (13)	0.0109 (13)	0.0019 (11)	0.0002 (11)	0.0002 (10)
N2	0.0181 (14)	0.0104 (12)	0.0089 (12)	0.0011 (11)	−0.0013 (11)	0.0010 (10)
N3	0.0247 (16)	0.0222 (16)	0.0132 (14)	0.0026 (13)	0.0005 (12)	−0.0002 (12)
C1	0.0149 (16)	0.0161 (16)	0.0119 (15)	0.0025 (13)	0.0015 (13)	0.0024 (12)
C2	0.0141 (16)	0.0124 (15)	0.0137 (15)	0.0031 (13)	−0.0031 (13)	0.0012 (12)
C3	0.0164 (16)	0.0138 (16)	0.0102 (15)	0.0023 (13)	−0.0043 (13)	−0.0014 (12)
C4	0.0161 (16)	0.0165 (16)	0.0130 (15)	0.0026 (13)	−0.0001 (13)	0.0002 (12)
C5	0.0193 (18)	0.0216 (18)	0.0228 (18)	−0.0006 (15)	0.0002 (15)	0.0013 (14)
C6	0.0213 (18)	0.0219 (19)	0.0257 (19)	−0.0073 (15)	−0.0045 (15)	−0.0028 (15)
C7	0.028 (2)	0.0192 (18)	0.0233 (19)	0.0012 (16)	−0.0072 (16)	−0.0064 (14)
C8	0.0209 (18)	0.0200 (17)	0.0151 (16)	0.0053 (14)	−0.0040 (14)	−0.0003 (13)
C9	0.0171 (16)	0.0146 (16)	0.0155 (16)	0.0040 (14)	−0.0040 (13)	−0.0005 (13)
C10	0.0164 (16)	0.0134 (16)	0.0164 (16)	0.0011 (13)	−0.0043 (13)	0.0010 (13)
C11	0.0193 (17)	0.0142 (16)	0.0118 (15)	0.0027 (13)	−0.0011 (13)	0.0008 (12)
C12	0.0185 (17)	0.0139 (16)	0.0129 (16)	0.0017 (13)	−0.0005 (13)	0.0035 (12)
C13	0.0262 (19)	0.0163 (17)	0.0133 (16)	−0.0008 (14)	0.0037 (14)	0.0011 (13)
C14	0.029 (2)	0.0248 (19)	0.0141 (16)	0.0014 (16)	0.0080 (15)	0.0073 (14)
C15	0.0218 (18)	0.0183 (17)	0.0202 (17)	−0.0032 (14)	−0.0012 (15)	0.0053 (14)
C16	0.0196 (17)	0.0172 (17)	0.0153 (16)	−0.0011 (14)	−0.0048 (13)	0.0011 (13)
C17	0.0167 (17)	0.0160 (16)	0.0140 (16)	0.0026 (13)	−0.0019 (13)	0.0030 (13)
C18	0.032 (2)	0.0218 (19)	0.026 (2)	−0.0113 (16)	0.0010 (17)	0.0001 (15)
C19	0.0142 (16)	0.0115 (15)	0.0162 (16)	0.0029 (12)	−0.0013 (13)	0.0014 (12)
C20	0.0197 (17)	0.0105 (15)	0.0131 (15)	0.0050 (13)	0.0012 (13)	0.0027 (12)
C21	0.0170 (16)	0.0168 (17)	0.0193 (17)	0.0009 (14)	0.0019 (13)	0.0003 (13)
C22	0.0238 (19)	0.0217 (18)	0.0173 (17)	0.0043 (15)	0.0102 (15)	0.0019 (14)
C23	0.030 (2)	0.0138 (16)	0.0140 (16)	0.0032 (14)	0.0037 (14)	−0.0026 (13)
C24	0.0226 (18)	0.0139 (16)	0.0129 (16)	0.0005 (14)	−0.0004 (14)	−0.0015 (12)
C25	0.0217 (17)	0.0103 (15)	0.0102 (15)	0.0029 (13)	0.0010 (13)	0.0021 (12)
C26	0.035 (2)	0.034 (2)	0.0175 (18)	−0.0041 (18)	0.0019 (16)	−0.0138 (16)
C27	0.0176 (17)	0.0192 (17)	0.0119 (15)	−0.0047 (14)	0.0012 (13)	−0.0020 (13)
C28	0.027 (2)	0.035 (2)	0.026 (2)	−0.0033 (18)	−0.0015 (17)	−0.0021 (17)
C29	0.0241 (19)	0.0248 (19)	0.0176 (17)	−0.0020 (15)	−0.0053 (15)	−0.0033 (14)
C30	0.031 (2)	0.031 (2)	0.037 (2)	0.0029 (18)	−0.0063 (19)	−0.0046 (18)
C31	0.0255 (19)	0.0165 (17)	0.026 (2)	−0.0014 (15)	−0.0012 (16)	0.0001 (14)
C32	0.026 (2)	0.025 (2)	0.026 (2)	0.0010 (16)	−0.0039 (16)	0.0008 (16)

### Geometric parameters (Å, °)

**Table:**

Co1—O1	1.990 (2)	C20—C21	1.419 (4)
Co1—O2	2.009 (2)	C20—C25	1.420 (4)
Co1—N1	2.101 (2)	C21—C22	1.371 (4)
Co1—N2	2.053 (2)	C22—C23	1.411 (5)
Co1—N3	2.033 (3)	C23—C24	1.376 (5)
Cl1—C28	1.745 (4)	C24—C25	1.430 (4)
Cl2—C28	1.770 (4)	C29—C30	1.498 (5)
S1—C27	1.635 (3)	C31—C32	1.508 (5)
O1—C17	1.316 (4)	C1—H1	0.950
O2—C25	1.315 (4)	C4—H4	0.950
O3—C16	1.380 (4)	C5—H5	0.950
O3—C18	1.433 (4)	C6—H6	0.950
O4—C24	1.380 (4)	C7—H7	0.950
O4—C26	1.436 (4)	C8—H8	0.950
O5—C29	1.499 (4)	C11—H11	0.950
O5—C31	1.499 (4)	C13—H13	0.950
N1—C2	1.419 (4)	C14—H14	0.950
N1—C11	1.287 (4)	C15—H15	0.950
N2—C3	1.430 (4)	C18—H18A	0.980
N2—C19	1.299 (4)	C18—H18B	0.980
N3—C27	1.166 (4)	C18—H18C	0.980
C1—C2	1.376 (4)	C19—H19	0.950
C1—C9	1.418 (4)	C21—H21	0.950
C2—C3	1.428 (4)	C22—H22	0.950
C3—C4	1.375 (4)	C23—H23	0.950
C4—C10	1.422 (4)	C26—H26A	0.980
C5—C6	1.374 (5)	C26—H26B	0.980
C5—C10	1.420 (4)	C26—H26C	0.980
C6—C7	1.414 (5)	C28—H28A	0.990
C7—C8	1.364 (5)	C28—H28B	0.990
C8—C9	1.419 (4)	C29—H29A	0.990
C9—C10	1.426 (4)	C29—H29B	0.990
C11—C12	1.448 (4)	C30—H30A	0.980
C12—C13	1.417 (4)	C30—H30B	0.980
C12—C17	1.420 (4)	C30—H30C	0.980
C13—C14	1.366 (5)	C31—H31A	0.990
C14—C15	1.404 (5)	C31—H31B	0.990
C15—C16	1.377 (5)	C32—H32A	0.980
C16—C17	1.428 (4)	C32—H32B	0.980
C19—C20	1.439 (4)	C32—H32C	0.980
			
O1···O5	2.766 (3)	O1···H30B	3.305
O1···C32	3.423 (4)	O1···H32B	3.483
O2···O5	2.745 (3)	O1···H32C	2.799
O2···C28	3.352 (4)	O2···H28A	2.417
O2···C31	3.580 (4)	O2···H30C	3.358
O2···C32	3.453 (4)	O2···H32B	2.804
O3···O5	2.931 (3)	O2···H32C	3.589
O3···C29	3.324 (4)	O3···H29A	3.028
O3···C31	3.410 (4)	O3···H30B	2.957
O4···O5	3.039 (3)	O3···H31A	3.059
O4···C29	3.424 (4)	O3···H32C	3.299
O4···C31	3.434 (4)	O4···H28A	2.732
O5···O1	2.766 (3)	O4···H29B	2.942
O5···O2	2.745 (3)	O4···H30C	3.558
O5···O3	2.931 (3)	O4···H31B	3.006
O5···O4	3.039 (3)	O4···H32B	3.389
O5···C30	2.464 (5)	H1···N3^i^	2.636
O5···C32	2.457 (4)	H28A···O2	2.417
C1···N3^i^	3.579 (4)	H29A···O3	3.028
C28···O2	3.352 (4)	H29B···O4	2.942
C29···O3	3.324 (4)	H30B···O1	3.305
C29···O4	3.424 (4)	H30B···O3	2.957
C30···O5	2.464 (5)	H31A···O3	3.059
C31···O2	3.580 (4)	H31B···O4	3.006
C31···O3	3.410 (4)	H32B···O1	3.483
C31···O4	3.434 (4)	H32B···O2	2.804
C32···Cl1^ii^	3.438 (4)	H32B···O4	3.389
C32···O1	3.423 (4)	H32C···O1	2.799
C32···O2	3.453 (4)	H32C···O2	3.589
C32···O5	2.457 (4)	H32C···O3	3.299
			
O1—Co1—O2	93.56 (9)	O5—C31—C32	109.6 (2)
O1—Co1—N1	87.90 (10)	C2—C1—H1	119.7
O1—Co1—N2	143.89 (10)	C9—C1—H1	119.8
O1—Co1—N3	108.59 (11)	C3—C4—H4	119.7
O2—Co1—N1	162.48 (10)	C10—C4—H4	119.7
O2—Co1—N2	90.36 (10)	C6—C5—H5	120.1
O2—Co1—N3	100.51 (10)	C10—C5—H5	120.1
N1—Co1—N2	78.44 (10)	C5—C6—H6	119.7
N1—Co1—N3	95.54 (11)	C7—C6—H6	119.7
N2—Co1—N3	105.92 (11)	C6—C7—H7	119.7
Co1—O1—C17	129.4 (2)	C8—C7—H7	119.7
Co1—O2—C25	127.9 (2)	C7—C8—H8	119.7
C16—O3—C18	116.6 (2)	C9—C8—H8	119.7
C24—O4—C26	116.5 (2)	N1—C11—H11	117.3
C29—O5—C31	112.7 (2)	C12—C11—H11	117.3
Co1—N1—C2	112.7 (2)	C12—C13—H13	119.6
Co1—N1—C11	126.9 (2)	C14—C13—H13	119.5
C2—N1—C11	119.9 (2)	C13—C14—H14	120.1
Co1—N2—C3	114.1 (2)	C15—C14—H14	120.1
Co1—N2—C19	125.6 (2)	C14—C15—H15	119.9
C3—N2—C19	120.3 (2)	C16—C15—H15	119.9
Co1—N3—C27	174.4 (2)	O3—C18—H18A	109.5
C2—C1—C9	120.5 (3)	O3—C18—H18B	109.5
N1—C2—C1	125.0 (3)	O3—C18—H18C	109.5
N1—C2—C3	114.8 (2)	H18A—C18—H18B	109.5
C1—C2—C3	120.2 (2)	H18A—C18—H18C	109.5
N2—C3—C2	114.9 (2)	H18B—C18—H18C	109.5
N2—C3—C4	124.8 (2)	N2—C19—H19	116.8
C2—C3—C4	120.3 (2)	C20—C19—H19	116.8
C3—C4—C10	120.6 (3)	C20—C21—H21	119.1
C6—C5—C10	119.9 (3)	C22—C21—H21	119.1
C5—C6—C7	120.7 (3)	C21—C22—H22	120.5
C6—C7—C8	120.5 (3)	C23—C22—H22	120.5
C7—C8—C9	120.6 (3)	C22—C23—H23	119.9
C1—C9—C8	121.7 (3)	C24—C23—H23	119.9
C1—C9—C10	119.5 (3)	O4—C26—H26A	109.5
C8—C9—C10	118.8 (3)	O4—C26—H26B	109.5
C4—C10—C5	121.5 (3)	O4—C26—H26C	109.5
C4—C10—C9	119.0 (3)	H26A—C26—H26B	109.5
C5—C10—C9	119.4 (3)	H26A—C26—H26C	109.5
N1—C11—C12	125.4 (2)	H26B—C26—H26C	109.5
C11—C12—C13	116.4 (2)	Cl1—C28—H28A	109.2
C11—C12—C17	123.0 (3)	Cl1—C28—H28B	109.2
C13—C12—C17	120.5 (3)	Cl2—C28—H28A	109.2
C12—C13—C14	120.9 (3)	Cl2—C28—H28B	109.2
C13—C14—C15	119.8 (3)	H28A—C28—H28B	107.9
C14—C15—C16	120.2 (3)	O5—C29—H29A	109.5
O3—C16—C15	124.3 (3)	O5—C29—H29B	109.5
O3—C16—C17	113.7 (2)	C30—C29—H29A	109.5
C15—C16—C17	122.0 (3)	C30—C29—H29B	109.5
O1—C17—C12	124.8 (3)	H29A—C29—H29B	108.1
O1—C17—C16	118.7 (2)	C29—C30—H30A	109.5
C12—C17—C16	116.5 (3)	C29—C30—H30B	109.5
N2—C19—C20	126.4 (3)	C29—C30—H30C	109.5
C19—C20—C21	116.3 (2)	H30A—C30—H30B	109.5
C19—C20—C25	123.9 (3)	H30A—C30—H30C	109.5
C21—C20—C25	119.8 (2)	H30B—C30—H30C	109.5
C20—C21—C22	121.9 (3)	O5—C31—H31A	109.8
C21—C22—C23	119.1 (3)	O5—C31—H31B	109.8
C22—C23—C24	120.3 (3)	C32—C31—H31A	109.8
O4—C24—C23	124.5 (3)	C32—C31—H31B	109.8
O4—C24—C25	113.2 (2)	H31A—C31—H31B	108.2
C23—C24—C25	122.2 (3)	C31—C32—H32A	109.5
O2—C25—C20	125.2 (2)	C31—C32—H32B	109.5
O2—C25—C24	118.0 (2)	C31—C32—H32C	109.5
C20—C25—C24	116.7 (3)	H32A—C32—H32B	109.5
S1—C27—N3	178.9 (3)	H32A—C32—H32C	109.5
Cl1—C28—Cl2	112.0 (2)	H32B—C32—H32C	109.5
O5—C29—C30	110.6 (3)		
			
O1—Co1—O2—C25	142.7 (2)	N1—C2—C3—N2	−2.6 (4)
O2—Co1—O1—C17	−178.3 (2)	N1—C2—C3—C4	179.5 (2)
O1—Co1—N1—C2	−166.2 (2)	C1—C2—C3—N2	176.7 (2)
O1—Co1—N1—C11	5.6 (2)	C1—C2—C3—C4	−1.2 (4)
N1—Co1—O1—C17	−15.8 (2)	N2—C3—C4—C10	−177.8 (3)
O1—Co1—N2—C3	88.5 (2)	C2—C3—C4—C10	−0.1 (3)
O1—Co1—N2—C19	−89.0 (3)	C3—C4—C10—C5	−179.7 (3)
N2—Co1—O1—C17	−82.8 (3)	C3—C4—C10—C9	0.7 (4)
N3—Co1—O1—C17	79.3 (2)	C6—C5—C10—C4	179.7 (3)
O2—Co1—N1—C2	−71.0 (4)	C6—C5—C10—C9	−0.7 (5)
O2—Co1—N1—C11	100.8 (4)	C10—C5—C6—C7	0.2 (5)
N1—Co1—O2—C25	48.4 (4)	C5—C6—C7—C8	1.0 (5)
O2—Co1—N2—C3	−175.0 (2)	C6—C7—C8—C9	−1.6 (5)
O2—Co1—N2—C19	7.6 (2)	C7—C8—C9—C1	−179.2 (3)
N2—Co1—O2—C25	−1.3 (2)	C7—C8—C9—C10	1.1 (5)
N3—Co1—O2—C25	−107.6 (2)	C1—C9—C10—C4	0.0 (4)
N1—Co1—N2—C3	18.6 (2)	C1—C9—C10—C5	−179.6 (3)
N1—Co1—N2—C19	−158.9 (2)	C8—C9—C10—C4	179.7 (3)
N2—Co1—N1—C2	−19.8 (2)	C8—C9—C10—C5	0.1 (3)
N2—Co1—N1—C11	152.0 (3)	N1—C11—C12—C13	168.5 (3)
N3—Co1—N1—C2	85.4 (2)	N1—C11—C12—C17	−12.5 (5)
N3—Co1—N1—C11	−102.9 (2)	C11—C12—C13—C14	−179.7 (3)
N3—Co1—N2—C3	−74.0 (2)	C11—C12—C17—O1	1.8 (5)
N3—Co1—N2—C19	108.6 (2)	C11—C12—C17—C16	−177.1 (3)
Co1—O1—C17—C12	14.8 (4)	C13—C12—C17—O1	−179.1 (3)
Co1—O1—C17—C16	−166.3 (2)	C13—C12—C17—C16	1.9 (4)
Co1—O2—C25—C20	−3.5 (4)	C17—C12—C13—C14	1.2 (5)
Co1—O2—C25—C24	178.4 (2)	C12—C13—C14—C15	−2.6 (5)
C18—O3—C16—C15	4.5 (4)	C13—C14—C15—C16	0.8 (5)
C18—O3—C16—C17	−177.5 (2)	C14—C15—C16—O3	−179.7 (3)
C26—O4—C24—C23	8.5 (4)	C14—C15—C16—C17	2.5 (5)
C26—O4—C24—C25	−171.8 (2)	O3—C16—C17—O1	−0.8 (4)
C29—O5—C31—C32	178.8 (2)	O3—C16—C17—C12	178.3 (2)
C31—O5—C29—C30	−172.5 (3)	C15—C16—C17—O1	177.2 (3)
Co1—N1—C2—C1	−161.2 (2)	C15—C16—C17—C12	−3.7 (5)
Co1—N1—C2—C3	18.0 (3)	N2—C19—C20—C21	−174.3 (3)
Co1—N1—C11—C12	6.0 (4)	N2—C19—C20—C25	3.3 (5)
C2—N1—C11—C12	177.2 (3)	C19—C20—C21—C22	177.8 (3)
C11—N1—C2—C1	26.4 (4)	C19—C20—C25—O2	3.9 (5)
C11—N1—C2—C3	−154.4 (3)	C19—C20—C25—C24	−178.0 (3)
Co1—N2—C3—C2	−14.6 (3)	C21—C20—C25—O2	−178.6 (3)
Co1—N2—C3—C4	163.2 (2)	C21—C20—C25—C24	−0.5 (4)
Co1—N2—C19—C20	−9.6 (4)	C25—C20—C21—C22	0.1 (3)
C3—N2—C19—C20	173.2 (3)	C21—C22—C23—C24	0.4 (5)
C19—N2—C3—C2	163.0 (2)	C22—C23—C24—O4	178.8 (3)
C19—N2—C3—C4	−19.2 (4)	C22—C23—C24—C25	−0.8 (5)
C2—C1—C9—C8	178.9 (3)	O4—C24—C25—O2	−0.6 (4)
C2—C1—C9—C10	−1.4 (4)	O4—C24—C25—C20	−178.8 (2)
C9—C1—C2—N1	−178.8 (3)	C23—C24—C25—O2	179.1 (3)
C9—C1—C2—C3	2.0 (4)	C23—C24—C25—C20	0.9 (4)

Symmetry codes: (i) −*x*+2, −*y*, −*z*; (ii) −*x*+3/2, *y*+1/2, −*z*+1/2.

### Hydrogen-bond geometry (Å, °)

**Table:**

*D*—H···*A*	*D*—H	H···*A*	*D*···*A*	*D*—H···*A*
C1—H1···N3^i^	0.95	2.64	3.579 (4)	172
C28—H28A···O2	0.99	2.42	3.352 (4)	157
C29—H29B···O4	0.99	2.94	3.424 (4)	111
C30—H30B···O3	0.98	2.96	3.607 (5)	125
C32—H32B···O2	0.98	2.80	3.453 (4)	124
C32—H32C···O1	0.98	2.80	3.423 (4)	122

Symmetry codes: (i) −*x*+2, −*y*, −*z*.
